# The dilemma of the symbols: analogies between philosophy, biology and artificial life

**DOI:** 10.1186/2193-1801-2-495

**Published:** 2013-10-01

**Authors:** Salvatore Spadaro

**Affiliations:** Department of Philosophy, Sapienza University of Rome, via Carlo Fea n.2, 00161 Rome, Italy

**Keywords:** Analogy-making, Connectionism, Theories of syntax, Genetic code, Artificial life, Cognitive robotics & AI, Binding problem

## Abstract

This article analyzes some analogies going from Artificial Life questions about the symbol–matter connection to Artificial Intelligence questions about symbol-grounding. It focuses on the notion of the interpretability of syntax and how the symbols are integrated in a unity ("binding problem"). Utilizing the DNA code as a model, this paper discusses how syntactic features could be defined as high-grade characteristics of the non syntactic relations in a material-dynamic structure, by using an emergentist approach. This topic furnishes the ground for a confutation of J. Searle’s statement that syntax is observer-relative, as he wrote in his book *"Mind: A Brief Introduction"*. Moreover the evolving discussion also modifies the classic symbol-processing doctrine in the mind which Searle attacks as a strong AL argument, that life could be implemented in a computational mode. Lastly, this paper furnishes a new way of support for the autonomous systems thesis in Artificial Life and Artificial Intelligence, using, inter alia, the "adaptive resonance theory" (ART).

## Introduction

Are symbols and symbolic languages natural or artificial (that is, independently created by humans)? This old philosophical "vexata quaestio" (discussed for example by William of Ockham) is still debated by scientists and philosophers in two specific areas: the use of the "computational metaphor" applied to the mind-brain in cognitive neuroscience and artificial intelligence (AI) and the synthetic way in which biological structures are reproduced in artificial life (AL). In AI and AL the inquiry focuses principally on the association with the essence of symbols, that is, objects materially constructed, according to definable and semantically understandable forms.

In classical AI the point in question about symbols has two faces, one concerning semantics and the other concerning syntax. The semantic part is the inquiry of how symbols, as syntactically characterized expressions of a particular kind, get their sense. The syntactic part is the question of how physical objects can also have syntactic meanings. As a theoretical and applied question in AI the semantic part of the argument has been designed by Stevan Harnad: the "symbol grounding problem" (Harnad 1990). This denomination is used, nevertheless, to talk about both the semantic and syntactic faces of the point in question. The symbol grounding problem includes, however, both a semantic grounding issue and a syntactic grounding issue.

In AL there are a lot of problems concerning symbols. The most important question has been detected by H. Pattee the "symbol–matter problem" (Pattee 1989). A practical example is: in a natural structure, how could a corporeal system work as a symbolic arrangement?

As said by C. Langton’s efficacious AL statement of belief (Langton 1989), AL tries to reply to the above problems by including life within a more general framework of possible life. In this way, the AL research concerns attempts to find the principles of constitution that denote life by imitating the properties of living entities on a computational structure. But Langton talks about another much discussed assertion of the AL manifesto, concerning the symbol–matter association. This type of the AL statement is known as "Strong AL", a name with some analogies with "Strong AI". (The latter expression was coined by J. Searle who criticizes AI’s positions [Searle 1980). If "Strong AI" says that an artificial agent can have mental states, "Strong AL" says that the aspects necessary for life are totally established, and so we cannot only imitate but also build living structures within an artificial system (Langton 1989; Bedau 2003).

The desideratum of this paper is to propose some useful analogies between the symbol–matter relationship in AL and the symbol grounding problem in AI. I will talk about the ideas of formal convention and syntactic definitions in relation to these issues considered in this paper. Another target is to show analogies between AL, the biological dominion, and the idea that symbolic labor in AI-inspired cognitive sciences is more effective than the idea of the formal classical approach.

## Against Searle’s conception of syntax

The typical symbol processing criterion in cognitive studies considers the cerebral operations as concerning symbolic calculations in an inner terminology of thinking and that the mind is, as a consequence, a sort of CPU. Searle’s famous argument against this theory is the so-called "Chinese Room Argument" (Searle 1980). This idea analyses the connection between the syntactic and semantic characteristics of intellective categories (for a general and historical introduction see Cordeschi 2002). In a more recent elaboration, Searle talks about the connections between the material and syntactic components of the mind (Searle 2004:91). In brief, this idea states that syntactic characteristics are intrinsically spectator-relative and this is impossible for a material structure, like the encephalon, and therefore it is impossible for it to have a syntax; so, the idea that a brain is a "syntactic motor" is not correct, according to this thesis.

The essence of Searle’s statement has three points, from which he draws a final interpretation:

1. Computational data processing is denoted syntactically as symbol manipulation.
2. The formal syntactic aspects of physical structures are constructed in regard to some codifying functions supported by a viewer outside the structure.
3. Consequently, a material structure is a syntactic, computational one and one only and is not inborn but associated to the mapping task. ("Syntax is not intrinsic to physics").

Thus, Searle concludes that subjects cannot consider a material structure as intrinsically computational but they can ascribe to it a computational form. Additionally, the syntax of a given structure is not the cause of our behavior.

It is significant to observe that this statement (explained in *"Mind: a brief introduction"*) differs from the Chinese Room Argument. The goal of the Chinese Room conjecture is the Strong AI point of view that the brain is a computational process, and the argument proceeds by showing that mental essence cannot be captured by the syntactic exploitations of computational processes.

In contrast to the above, this paper argues in favor of what Searle calls (quite ironically, in my opinion) "Cognitivism", that is: the mind is a computational machine and therefore attempts to show that "syntax" is not basically an observer-related assumption ("Cognitivism" considers syntax as an intrinsic feature of the matter). If Searle’s ideas were right about the connection between material and syntactical structures, the consequences for AI and AL would be substantial: the idea that the mind is a syntactic machine would be incongruous; and, the idea that mental mechanisms are computational would have no link to the ground. And Strong AL too would be indefensible, because it is irrational to say that there could be a viewer-absolute computational midway in which to construct the principles of living beings.

Although Searle’s idea on syntax is, in a certain way, connected to his view on the philosophy of mind, his assertion that "syntax is not intrinsic to physics" could be controverted without getting involved in the philosophical discussions about intentionality and qualia. First of all, we can bypass Searle’s puzzling separation between epistemology and ontology by assuming that, as in the scientific tradition, problems regarding what something is, could not be discussed independently from problems referring to how we discover what something is. In the current discussion, this could signify that the statement regarding what syntactic characteristics are, cannot be studied independently from the question of how a structure gets syntactically explicated. From this point of view, the parallel "syntax/semantics" could be exhaustive: only when we discover that a structure is semantically knowable, we don’t require further questions regarding if it is "really" semantic (see Haugeland 1997); similarly when we find that a structure is syntactically understandable we don’t require further investigation regarding if it is "really" syntactic. However, "really" (or "epiphenomenologically") doesn’t obligatorily correspond to "intrinsically" (or "essentially"). A thing could be really X without being intrinsically X; it could be F only "interrelationally"; for example in Hobbes’ second of the sixteen "Objectiones ad Cartesii Meditationes" (1641) he said, talking about the mind, that it is not correct to argue from "I am thinking" to "I am thought’ or from "I am walking" to "I am a walk": an intelligent thing is not the same to intellection (Hobbes used, I think, a materialist point of view but this example is also very interesting in an emergentist context). Alternatively, we should examine the theoretical and experimental reasons used in defense of the syntactic ground in computational projects such as AI or AL. The next two sections will be dedicated to this chore and they will affirm that there are strong reasons to confute Searle’s affirmation that "syntax is not intrinsic to physics".

The strategy used will have some significant effects. It will support the position that Searle is de facto disputing against—the cognitive approach; however, it will amplify some problems regarding the relevance of the Strong AL argument. It could also produce a new procedure to reinforce the "complex autonomous system" research agenda within AL, connectionism and cognitive science (Varela & Bourgine 1992; McClelland 2010). From this research activity a more effective idea of symbolic working will, hopefully, be forthcoming.

## An intrinsically syntactic mind

We could start by examining the classical cognitive thesis for determining the mind, at a determined level of analysis, as a material symbol system. Two principal theses have been furnished. The first one addresses intentionality, particularly to the semantic states of mental categories that are involved in the production of behavior and that we believe are envisaged in the mind. The second one addresses complexity, in particular to the structural complexity of the mind.

 In his books *"Computation and Cognition"* (1984) and *"Things and Places"* (2007), Pylyshyn talks about how computational descriptions must be considered in relation to the measuring supported by two interpreting operations, the semantic function (SF) and the instantiation function (IF). On one hand, The semantic function codifies from distributed functional states onto some spheres of specific interpretation. This process is necessary for formal procedures as a calculation because it is a rule-governed modus operandi characterized beyond semantic aspects (Fodor et al. 2002). For this reason, the first assumption of Searle’s thesis, that computation is described only at the syntactic level, isn’t very correct within the ambiance of the symbol processing procedure.

On the other hand, the instantiation function connects from material categories to computational categories.

This is the interpretation function which is in accordance to the interpretation of syntax: its mission is, therefore, to demonstrate how syntactic elements are physically constructed. Unfortunately, Pylyshyn’ s analysis of the instantiation function doesn’t supply many elucidations about this matter because the syntactically different elements calculated in the evaluation aren’t easily ascribed to the structure, but intrinsic in it. The question becomes more complex when it is said that computational elements are independent from any determined physical medium; however, multiple realizable material productions of a computational function, as stated by Pylyshyn, are principally open-ended.

So multiple realizability is usually recognized as a positive element in the computational metaphor of the brain, for it supplies a representation of how not strong token-token identities are required between brain cases and material cases.

Searle answers, per contra, that the multiple realizability of computational cases is only an evidence that the mental properties are not inherent to the structure, but derived from an external analysis. He observes that a differentiation should be given between tools whose purposes are multiply realizable (like, thermostats) and are, however, described as the creation of identical material effects (like, the regulation of temperature), and tools whose multiple realizability is expected from their essentially formal characteristics (for example, Turing machines).

This differentiation between what could be denominated functional multiple realizability and formal multiple realizability signifies the existence of another important idea. This is the idea of digitality. One argument that is usually furnished for explaining why material symbol structures could be multiply realizable is that they are digital structures. It is not very clear among cognitive scientists or philosophers how to describe digital structures and their functions as, e.g., processing information (Pylyshyn 1984; Chomsky 2004), but for our purposes, a digital structure is one whose actions are part of a limited number of categories that are always determined; however, for any given category, a case is either of that sign or it isn’t, and difference among the cases that appertain to a certain type isn’t significant. In material symbol structures, the irrelevant differences appear at the material level and the well determined types harmonize with the syntactic characters of the structure. In this manner syntactic characters accord freely with a lot of material properties and syntactic case-transitions could freely include many material causal rules. However, material symbol structures are, as a group, unconnected to any determined physical medium.

The other argument, that we will explain, concerns the organizational complexity of the mind.

Melanie Mitchell (2009) explains the thesis of organizational complexity in her book: *"Complexity: a guided tou*r". Mitchell says that computational grades of description are required for structures whose elements associate combinatorially. In the computer, the status of each element (for example binary switches) is free from the statuses of the other elements; however, the activeness of larger elements in the machines isn’t a simple sum, but counts on the state of each element and their intrinsic combinatorial characteristics. The complexity of the combinatorial characteristics is basically accountable for the medium-independence of machine structures.

The precursor for this thesis about the mind is of course the crucial paper by Warren McCulloch and Walter Pitts (1943) entitled "A Logical Calculus of the Ideas Immanent in Nervous Activity". By considering the neurons as the functional elements of the nervous system and by interpreting neuronal activeness as binary, digital, and simultaneous (all change status at equal distinct time-steps), McCulloch and Pitts were capable to demonstrate that the organizational complexity of a certain number of neural networks is adequate for the computing of the formal logic processes (propositional calculus). This development has been very important for the entire area of cognitive sciences because it explains how the processes of the nervous system could be defined, at certain levels, using formal logic and , therefore, how the mind can be described as a symbolic machine; moreover, following recent studies (Volterra & Meldolesi 2005; Allen & Barres 2009) also glial cells could have a relevant role within the brain working and thus in cognition; they usually are smaller than neurons and outnumber them by five to ten times; they include about half the whole mass of the brain and spinal cord, there are various kinds of glial cells, for example, astrocytes, oligodendrocytes and so on; in the past their role has been undervalued but, probably, they have a key role in organizational complexity of the brain and it would be useful a development of a "gliascience" (glial cells differ from neurons) in biopsychology and a "computational glia-neuroscience" in AI (integrating artificial neural networks and artificial glial networks).

The organizational complexity allows the beginning of a more efficient analysis of the syntactic interpretability themes. Assume, for example, that McCulloch and Pitts’s idealized neural networks attested the real functional structure of the mind. It would still be true that in designing a computational structure of the mind, we would have to reproduce the all-or-none activity of the neuron onto 0′s and 1′s in binary script, but the structure would be, in a case, non-arbitrarily based on the intrinsic characteristic of the mind, that is, the function that neuronal activity performs in the actions of the nervous system and the production of behavior. Searle considers this question, but he simply declines it by repeating his statement that "syntax is not intrinsic to physics", avoiding the entire issue.

My reference to McCulloch and Pitts should not be considered as an affirmation about how computational operations are really based on mind operations. To the contrary, it is recognized that real neurons aren’t only binary switches, and even though neurons are the material units of the nervous structure, the essential functional units are presumably relatively unvarying patterns of activeness in neuronal aggregations (Edelman et al. 2000). However, by citing McCulloch and Pitts I would like to show the point that, against Searle, there is nothing discordant in assuming that computational mechanisms could be based on the organizational complexity of nervous structures. It’s for this reason that I thought the issue of organizational complexity supports the development of a way to syntactic interpretability: It critically replies to Searle’s theoretical thesis, and, at the same time, leaves open the empirical questions about the syntactic interpretability of complex structures like the mind. The approach to the thesis that I have followed could have theoretical connections for these empirical questions. We have comprehended that for the ground properties of syntactic interpretability it isn’t sufficient to only invoke the ideal correlation of the syntactic to the material, such as Pylyshyn’s IF; we should also refer to the organizational complexity of the producing structures. For this reason, I believe that Fodor and Pylyshyn (1988) are wrong when they say that philosophers such as Hofstadter, Dennett and connectionists such as Rumelhart and McClelland (1986) should not invoke the complexity as a line of differentiating between cognitive and non cognitive structures. Instead, it’s the organizational complexity of determined structures that authorizes the idea that there are other syntactic grades of description for their behavior.

The new research field recognized as emergent computation is very hopeful (Bertelle et al. 2006). Emergent computation is the analysis of complex structures having three general characteristics: (1) they are made of a group of agents each of which replaces explicit rules; (2) the agents interact following the rules and thereby generate implicit, emergent general designs; and (3) there is an interpretation function that connects the general designs onto computations. In these emergent computational structures the low-grade agents are themselves tools that have only a formal specification, but since they are usually plain—e.g., the on-off cells of a cellular engine—we could clearly conceive biological analogues.

A good biological example could be the mirror neurons, recently discovered by Rizzolatti and his research team (Rizzolatti et al. 2004). These neurons fire both when an animal acts and when the animal looks at the same action performed by another one. Thus, the neuron "mirrors" the behavior of the other, as though the observer were himself acting. Such neurons have been directly studied in primate and other species. In humans, mirror neuron activity has been recognized in the premotor cortex, the supplementary motor area, the primary somatosensory cortex and the inferior parietal cortex (Kohler et al. 2002). I will call this sort of mechanism "Neuro-Motor Analogical System" (NEMOANSY) because it is situated almost in the motor cortex and it is based on a analogy-making model that is considered a very important tool of intelligence: the cognitive scientist and philosopher D.R. Hofstadter said analogies are the core of cognition (Hofstadter 2001). Moreover, to understand the actions of others it is necessary not only a inner syntax but also the sharing of it between agents (humans and non-humans).

## Biological symbols

A good example of a structure that has a syntactic grade of operation in this context is given by living cells. De facto cells represent a minimum, emblematic exemplification of a structure that is independent (this point will be made clear later) and naturally operational on a syntactic grade, therefore, I will continue discussing them.

In cells the syntactic grade can be certainly assimilated to the so-called "genetic code" whereby genes (the DNA) indicate the types of proteins a cell can produce. More correctly, the "genetic code" alludes to the laws that assign characteristic amino acids to the characteristic triplets of the nucleotide bases in DNA. Protein synthesis, therefore, includes specifications that are written in DNA and then "deciphered" by a complex mechanism including molecular transcription as the production of mRNA by RNA polymerase and nucleotides, and "translated" (production of protein by mRNA, ribosome, etc).

The relationship of specification between DNA and proteins has a certain group of characteristics that legitimize, utilizing the syntactic term "code", its explanation. First of all, the genetic code is quasi-universal: with the exception of mitochondria, nucleotide triplets describe each time the same amino acids indifferent to what the living being is; for example, the triplet AAG (adenine–adenine–guanine) designates lysine in all the organisms from a bacter to a human being. Secondly, the code is arbitrary in the sense used by Maynard Smith who said it is difficult to explain why a code in which GGC signifies glycine and AAG signifies lysine is more appropriate or defective than one in which the significances are reversed (Maynard Smith 1989). Thirdly, the code is compositional, namely, there are a lot of chances for nucleotide triplet compositions in a linear pattern. Fourthly, the code is digital because what a nucleotide triplet counts on its sign out of a limited number of classes and material variations within determined limits among tokens of a given class making no difference to their being part of that class (Ganti 2003).

These four characteristics: quasi-universality, arbitrariness, compositionality, and digitality allow the explanation of the genetic code as including syntactic connections. But the genetic code doesn’t count on an interpretation function given by an outer spectator; neither it is a set of rules applied by a control that is external to the cells. In a certain degree, it is a set of conditions put in the metabolic processes that establishes the cell as an individual. To give this point its right accent in this context, I would use Howard Pattee’s interpretation of the connection between the material and the syntactic aspects in biological structures (Pattee 2001). Pattee distinguishes the rule of nature themselves which are universal and closed (holonomic) from systems that conform to the rules of nature and further limit the movement of matter (non-holonomic systems). In Pattee’s viewpoint, the existence of such supplementary architectures in a structure could ground its syntactic interpretability: we connect the structures onto syntactic elements, which can be explained without designating the material structures realizing them.

We can now consider how DNA, as a natural syntactic structure, is and must be inserted into the inner workings of the cells. Generally, nucleotide triplets are able to specify themselves in amino acid if and only if they are properly included in the cells’ metabolism, for example, in the myriad of enzymatic modifications in an intricate chemical net. This network has a "chicken and egg" nature at different levels. Firstly, proteins can proceed only from a decoding procedure, but this procedure itself can’t occur without proteins; and secondly, the protein decodification and construction procedures must be accurately located within the intracellular environment, but this ambiance is itself a consequence of those very procedures (Maturana & Varela 1987). Therefore, when we refer to DNA coding for proteins we are not indicating a determined kind of syntactic causal connection; we are, rather, concentrating a long constant causal sequence of physical and biochemical occurrences. It is exactly the constancy and predictability of all sequences that ground on nucleotide triplets as symbols that are valid for amino acids.

Therefore, the genetic code can be used as a counter example to Searle’s position that syntax is basically relative to the spectator. This can be right, only with a determined specification. Although I am claiming that it is admissible to see the specification relationship between DNA and proteins on a symbolic level, I am not claiming that we could overlap the software-hardware differentiation from symbolic computation onto the biological cells. E.g., we could examine a declaration from F. Dyson’s book *"Origins of Life"*; he said that hardware evaluates information; software incorporates information. These two elements have their analogues in biological cells; proteins are hardware and nucleic acid is software (Dyson 1985). But protein and nucleic acids are not "exactly analogous" to hardware and software levels. The connection is not very correct because although there is a legitimate point in which the "self-description" of the protein-design of the cells included in DNA could be explained formally, it must be considered that the concept "self description" is a shortcut to refer to connections that must be dynamically incorporated. There isn’t a specific analogy to this dynamical incorporation in software.

Now, we can see that the description of syntactic interpretability explained by the case of DNA in living cells is strongly favorable for the material symbol structure model of the mind, which is contrasted by Searle’s thesis. This model plainly takes symbols at apparent value and uses them as if they were free from the neural processes on which they are based. Considering living cells as a structure that has a biological syntactic grade of processing, we can say that there isn’t a complete independence of the symbol substrates from the dynamical environment in which they are inserted. This point has significative consequences for the position of "Strong AL" and for the AI debate between connectionism and the symbol processing model.

## Bound symbols

In this paragraph I will try to explain how these symbols, intrinsic to natural and also artificial structures, are integrated with each other, in reference to the so called "binding problem". The binding problem is, firstly, an interdisciplinary question analyzed in cognitive sciences, philosophy of mind, biology and Al (in particular in the connectionist approach). We can define it by saying that we perceive the outer world where we live as constituted by complex objects that are compounded by their colors, shapes, movements and so on. To recognize an object, we must determine not only its parts and properties, but also how those parts and properties are combined. We want to study what workings allow us to perceive the correct conjunctions, for example a pair of blue jeans as blue and a white T-shirt as white and not the reverse.

One of the most interesting theories about this question is the so called "FIT" ("Feature Integration Theory") by Anne Treisman (Treisman 1999). This model analyzes three distinct spatially selective procedures to solve the binding problem: selection by a spatial attention window, suppression of locations from feature maps comprehending undesired features, and top-down activation of the location comprehending the focused on objects. The "window of attention" checks a master map of areas, selecting the characteristics active in corresponding areas of some specialized feature maps. These features can be considered as tokens or symbols because the binding problem is present also in words and sentences. For example some experiments have established that readers are deeply perceptive to multi-letter units of analysis (Lima et al. 1983). The binding problem is also discussed by connectionists that describe it using two groups of binary units for codifying the position of a point in a two-dimensional space. The units with activation in the X and Y groups depict the x and y coordinates (Rumelhart et al. 1986:88-90). The binding problem will emerge if two points must be encoded at the same period of time, it isn’t possible to say which X coordinate goes with y coordinate.

My idea is that, both in biology (in particular in genetics) and in AL, symbols are not only intrinsic to matter but they interact and integrate with themselves.

Can one symbol be isolated? As explained by the philosopher and cognitive scientist Douglas Hofstadter, this is probably not possible because symbols, in the world, are always connected to other symbols; he used the example of males and females of a determined species that are always together (Hofstadter 1979:359). Moreover, I add that symbols of a determined type are not only useful but necessary to comprehend the characteristics of another type of symbols.

However, the binding process is always constituted by interactions of symbols, that are *all* necessary (I will clarify why I emphasize the term "all"). As we have seen the DNA code is a good example of how syntax is inside matter but it is necessary to specify how single genes are bound. One method to comprehend the binding is to consider DNA as a matrix of symbols (an approach used also in AL and AI) that represent the binding sites: there is a matrix symbol for all possible positions in every site; the result for every site is obtained computing the sum of matrix values for a determined sequence of a specific site. Professor G. Stormo has elaborated a "binding rule" for DNA code (Stormo 2000:19) that could be interpreted as a syntactic rule because it describes the formal behavior of these symbols interaction and binding. However, this thesis could be invalidated if we consider that about 99% of DNA is non-coding and named by Susumo Ohno "Junk DNA" (Ohno 1972). I think that this name is totally inappropriate because, adopting the traditional Ockham razor in genetics, these genes should be eliminated by evolution if they are really useless and moreover the inner syntax, that we are defending, wouldn’t consider the majority of the symbols (the genes).

But recent studies of the "ENCODE" project (published in September 2012) have demonstrated that over 80% of "junk DNA" participates in a variety of biochemical purposes (Pennisi 2012). My opinion is that every gene participates in some processes and interacts, binding to others, because the interpretability of a symbol, as I tried to explain, is connected to *all* the symbols of a determined language (biological or artificial) and syntactic rules should be valid for all the elements of the language itself.

## Artificial life and functionalist theories

As reported by Christopher Langton and Thomas Ray, "Because we cannot observe life on other planets, we are left with the alternative of creating Artificial Life forms on earth. We will discuss the approach of inoculating evolution of natural selection into the medium of the digital computer. This is not a physical\chemical medium; it is a logical\informational medium" (Langton 1995:179). The framework of this observation is a consideration on machines, and how, in the evolution of cybernetics and the formal theory of computation, "The 'logical\informational medium’ of a machine is divided from its physical support of construction, and we discover that "to be a machine" is a characteristic of the former, not of the latter.to make this thesis correspond to the same idea in the case of living beings, it is necessary to make a supplementary premise that organisms practically are a kind of machine; the type of machine could then be delineated by a peculiar structure (see Fontana et al. 1994).

In philosophy this thesis is common not so much in debates on life but in debates on mind and is known as functionalism (Putnam 1975). The conviction is that the logical form of a mind can be separated from its physical supports, and that mentality is a character of the former, not of the latter. The functional aspect of a state is something conceptual in the sense that it can be determined as a group of relationships without referring to the physicality of the states that happen to incorporate those relationships; and any physical element that can sustain the appropriate network of relations will be sufficient to actuate the functional role. However, the multiple realizability of mind comes from metaphysical functionalism. When metaphysical functionalism is connected with what is often called "computation-representation functionalism", the idea that, using a psychological definition, mental states could be described as constituent algorithmic processes delineated over symbols after the configuration of a computer program; we reach the viewpoint that it is possible to design minds in a formal computational (symbolic) medium (Strong AI) but it is not very clear how physical and logical form can be totally divided in an living being.

Strong AL could be the computational side of metaphysical functionalism referring to the biological class of life in place of the psychological class of mind. Strong AL assumes that what makes a state a "living" state (involved in, for example, metabolism) is plainly its functional aspect. However, the logical configuration of a living being—the body of functional relations containing all its constituent states and processes—can be appointed without alluding to the living entity’s physical structure; and the physical structure can be anything as long as it can sustain the correct class of functional relations. So, multiple realizability accords with the biological sphere. Strong AL thesis also assumes, that the logical form of a living being can be understood totally in a symbolic representation, and so we come at the viewpoint that life is possible in a logical and computational medium. Thus, there is a distinction in the kind of computational way taken in Strong AI and Strong AL. In computation-representation functionalism, mental modus operandi is supposed to be recursively analyzed in a top-down way, whereas AL biological modus operandi is supposed to be recursively obtained in a bottom-up way. But this difference doesn’t deal with the most important element, which is that the target area of epiphenomena in each occurrence (mind or life) is recognized to be obtainable in a computational medium.

So, the inquiry that needs to be answered is if it is really possible to abstract the logical configuration of a living being from its physical structure in the form of a symbolic definition that could also be a realization of a living structure, in estimating strong AL. The discussion in the previous section suggests this may be impossible. Recall Pattee’s (2001) difference between the rate-independent linguistic manner and the rate-dependent dynamical manner in cellular activities. An important point connected with this differentiation is relevant here; as Pattee says, the transduction from the first way to the second is not itself linguistically explained, but, in a certain degree, is realized by the dynamic interaction of the cellular elements following the rules of nature.

This interconnection of corporality and form in the cells has also been considered by Emmeche (2004). He says that the timing of the mechanisms is critical in both the translating of DNA into mRNA chains and in the synthesizing of enzymes, from amino acids to the ribosomes (transcription). The composition is administered by an "attenuation control structure" that includes both the linguistic way (protein coding) and the dynamical way (the material form of the RNA). For this reason, Emmeche criticizes Pattee’s linguistic-dynamical differentiation: "Pattee (1989) was emphatic about the distinction between a model of life and a realization of some life process […]. Considering the possibility of a 'wet’ bottom-up synthesis of other forms of life, we need to expand the kind of analysis given by Pattee to include not only the role of computational models in science in general and Artificial Life in particular, but also the very notion of a model in all its variety, and especially the notion of model organisms in biology." (Emmeche 2004:122). Anyway, the element I desire to extract from these statements is that, since the synchronization of transcription and translation proceedings in the cells is very important, the logical shape of the cells, as a dynamical structure, isn’t atemporal and absolute (it is a feature of abstract, symbolic figures), but quite time-dependent. For this motif, substance and form may not be divisible in biological structures such as in living beings.

The general question about Strong AL is how it imagines the connection between substance and form in the biological context. Langton states life comes from a specific type of pattern, not substance, an effect of the organization of substance (Langton 1995). According to this statement, life can be interpreted as an emergent process dependent on phenomena having a typical shape or organization (Fontana et al. 1994; Ruiz-Mirazo et al. 2000). But there is something wrong following this interpretation: in the biological context at least, shapes are something, as Aristotle enunciated long ago, that cannot be separated from substance itself. Eric Karsenti uses this sentence correctly when he says: "The problem is that embryogenesis and dynamic cell forms and functions emerge from multiple molecular interactions and interconnected regulatory feedback loops" (Karsenti 2008:255).

It is useful to show that the reasoning just presented doesn’t avoid to applying the thesis of multiple realizability *per se* to living beings. So, what is contested here is the stronger conception of complete computational realizability for living structures. Put differently, none of the observations advanced so far exclude the eventuality of life being realizable in many diverse material media (Searle’s conception of physical multiple realizability).What the arguments talk about is the theoretical intelligibility of the notion of purely computational life (Searle’s idea of theoretical multiple realizability as enforcement to life). Analogous conclusions were achieved by S. Harnad in his reasoning on symbol grounding and Artificial Life (Harnad 1994). Harnad says that a computational structure in general is a semantically non symbol based structure; it gets grounded only when provided with interpretations. However, a computational pattern of life cannot itself be living: even if the pattern proves to be formally like a living being (if this is its precise meaning). It is, however, only relevant to an interpretation, relevant to some semantic features that connect the symbols of the pattern onto parts of living mechanisms. A current living being, however, though it could involve symbolic mechanisms of different kinds, isn’t itself an ungrounded symbol structure.

As Harnad demonstrates in his argument, he is prepared to consider that *every* essential element for life could be systematically reconstructed in an artificial pattern. Hence, I am a little skeptical of this thesis. If, as Pattee and Emmeche say, the logical structure of living cells has a time-dependent side (for example, having to do with times of reaction), then it is improbable that the logical structure (or form) of a living being can be totally reconstructed in a purely computational structure. I support this thesis provisionally because a solution of the problem in one way or another needs a fully expanded theory of the structure proper to life. The study of constructing such an explanation has certainly begun (Fontana et al 1994; Ruiz-Mirazo et al. 2000), but remains to be accomplished.

## Analogies with artificial intelligence

The previous argument also has consequences also for the discussion in AI between connectionism and the symbol processing thesis. As observed above, the cognitive approach of the mind as a material symbol structure takes the symbolic grade for granted and uses it as if it were autonomous (in theory) of the neural mechanisms within which it is assumed to be obtained. In the case of biological structures such as the cells, there isn’t such independence of the level of symbolism from the besetting dynamic environment. Excluding the biologically unconstrained, top-down theories founded on standards of mental representation, there aren’t any reasons against assuming the same conditions hold for the various kinds of formal regularities to be discovered in the mind and nervous structures. However, it is harder to specify the mechanism accountable for these regularities in structures that are organizationally intricate. This difference in the complexity of the system doesn’t legitimize the indifference that the symbol processing approach usually shows to the dynamical framework of symbolic action.

The analysis of the symbol processing modus operandi is nothing new to those who are involved in the connectionist research project (Rumelhart & McClelland 1986; McClelland 2010). Connectionist models don’t take symbols at par value and make entirely top-down considerations about the construction of symbol structures in the mind. Alternatively, symbols are usually treated in the connectionist method as approximate macro-level condensations of operations whose governing rules reside at a "sub-symbolic" grade (Hofstadter 1979; Rumelhart & McClelland 1986). This general kind of connections between the symbolic and sub-symbolic grade holds for the DNA in living cells: Delineating nucleotide triplets as "coding" for amino acids is to shorten a lengthy causal catenation of complex intracellular processes whose controlling laws reside at a sub-symbolic biochemical grade. However my use of the cells as a basic criterion of how syntax could be intrinsic to physics turns out to support considerations in reinforcement of connectionism.

The word "connectionism" is of course typically used for patterns of sub-symbolic principles in neural nets. The target here is both to comprehend biological neural nets and to solve questions in the theory of machine learning. Moreover T. Maia and J. McClelland (2005) said that the word "connectionism" should be given a much broader meaning. They interpret a connectionist model as one in which the relations between the variables at any stated time are restricted to a limited number of connections, and the weights of the connections can modify in time. They then show that this group of (meta) dynamic structures includes not only neural nets, but also classifier structures in AI, immune networks, etc.

With connectionism interpreted so broadly, the most relevant concern of the discussion is the theory of autonomous systems (Varela & Bourgine 1992; Rehtanz 2003). The most important distinction here is between heteronomous structures, which are described by input-output functions and an outer control, and autonomous structures, which are determined by internal mechanisms of self-organization. F. Varela has tried to make this distinction more precise by stating that the mechanisms that make up an autonomous system must (1) be connected as a network, (2) generate and produce themselves, and (3) constitute the structure as a whole in the dominion in which those mechanisms exist. Varela reassumes this thesis of autonomous systems as self-constituting nets of mechanisms in what he names the "Closure Thesis," which says that all the autonomous systems have operational closures (Varela & Bourgine 1992). The word "closure" is utilized here in its algebraic meaning: a given dominion has closure if all operations determined in the dominion remain within the same dominion. However "operationally closed" in this framework doesn’t signify that the structure is materially and relationally closed to the outside, but quite that inner and outer are obtained by the self-constituting dynamics of the structure itself.

An important example of a structure that is claimed to be autonomous is the nervous system. The thesis of the nervous system as an autonomous rather than heteronomous structure is correct, relative to connectionism and its ratio with the dynamical framework of symbolic activeness. E.g., within neural network research structure whose learning is totally "supervised" and ascribed as heteronomous because it modifies the connections in the network that are managed by an external training sign, as in the learning algorithm known as "backpropagation" (Rumelhart & McClelland 1986). Backpropagation can’t be described without any concern to such a training sign that is outer and independent of the structure; however, back-propagation connectionist structures cannot be autonomous. Conversely systems whose learning is "unsupervised" catch a key side of autonomy because their exchange to their relations in the network usually depend on cooperative and competitive connections between the nodes without the leadership of any external supervisor (Carpenter & Grossberg 1990; Smith 2002).

However, in this type of autonomous neural network study, one of the most important goals is to comprehending how symbolic mechanisms emerge in the mind. One promising case is the "adaptive resonance" neural network thesis of S. Grossberg, G. Carpenter, and their associate.s (Carpenter and Grossberg 1990). Their ART (adaptive resonance theory) and more recent ARTMAP models utilize unsupervised learning rules, but they can also work in a supervised approach when there is feedback from the ambiance. The models fit competitive learning rules in self-regulating control systems hold both attentional and directional substructures. The relationships between these two substructures allow the network to self-organize in real time solid inner configurations in reaction to arbitrary strings of arbitrarily input schemes without any previous explicit representation of the ambiance. Grossberg and Carpenter name a certain group of solid, inner configurations a "recognition code"; the symbols that constitute the code are compressed, yet they are created and stabilized through mechanisms of resonant binding that are distributed across the structure (Carpenter & Grossberg 1993). These detections have been recently used to create artificial neural networks with mirror system (NEMOANSY). The BiARTMAP (Butz et al. 2003), using an evolution of the "Adaptative Resonance Theory", associates achieved actions with resultant action-effects. Mirror capabilities are given by the associative structure. On the one hand, detected environmental changes establish action associations. On the other hand, stimulated action models create the apprehension of resulting environmental change. The BiARTMAP approach is also used in the ICub robot: it has the size of a three year old baby and is able to recognize and manipulate objects with its hands. This "baby" robot acts in a cognitive scenario, using a hybrid structure (symbolic and sub-symbolic), performing tasks useful to learning while interacting with the environment and humans (Marocco et al. 2010). This robot is built by adopting the strategy of "biological compatibility": the subject acts in a complex environment, learning how to behave in new situations.

Adaptive resonance theory, however, furnishes a model of how solid formal configurations, important for the perception and action, can emerge as an outcome of distributed subsymbolic mechanisms and then act to frame the adaptive behavior of the structure. Moreover, it furnishes the right kind of model to comprehend how syntax can be inner to physics in the ambit of neural and cognitive mechanisms. (Using "right kind" I mean one that satisfies the requisite stated in the third paragraph of addressing how formal orderliness can emerge as an effect of a system’s autonomous working while also serving for what the system could do.)

The autonomous systems study project in AL and AI appears, however, to be in a fine position to argue that it can be able to find the rules of "qualitative structures" below symbolic activity in complex patterns. This thesis of a rule of qualitative structure was originally demanded in cognitive sciences by Newell and Simon (Newell & Simon 1977) on behalf of their material symbol structure ideas. As we have seen, however, the merely top-down method that this idea takes toward symbol grounding is not satisfactory. Conversely connectionism and in particular the ism of autonomous systems regards the dynamical ambiance of symbolic activeness, and thus hold the promise of a formal "ism" of the necessary and sufficient conditions for symbolic activities both in natural and artificial dynamical structures.
